# Malaria situation in a highly endemic province in the Central Highlands of Vietnam: a retrospective analysis

**DOI:** 10.1186/s12936-026-05896-y

**Published:** 2026-04-07

**Authors:** Thang Nguyen-Tien, Vu Tran, Tham Minh Nguyen, Phuc Hai Hoang, Dat Minh Le, Richard James Maude

**Affiliations:** 1 Mahidol-Oxford Tropical Medicine Research Unit, Faculty of Tropical Medicine, Mahidol University, Bangkok, 10400 Thailand; 2 Vietnam Public Health Association, Hanoi, Vietnam; 3 Department of Internal Medicine (Infectious Diseases), The University of Texas Medical Branch at Galveston, Galveston, TX USA; 4 The University of Queensland, Brisbane, QLD Australia; 5 University of Canberra, Canberra, ACT Australia; 6 Dak Lak Centre for Disease Control, Buon Ma Thuot, Dak Lak Vietnam; 7 Centre for Tropical Medicine and Global Health, Nuffield Department of Medicine, University of Oxford, Oxford, Oxfordshire UK; 8 The Open University, Milton Keynes, UK

**Keywords:** Malaria incidence, Malaria risk, Central Highland Vietnam, Retrospective analysis

## Abstract

**Background:**

Vietnam is progressing towards nationwide malaria elimination by 2030 with residual transmission limited to a few provinces in the Central region including Dak Lak. These areas, especially in the central highlands, continue to have challenges with ongoing forest transmission, movements of vulnerable populations, monitoring and management of imported cases, drug resistance and financial sustainability. This retrospective study aimed to understand the malaria situation in Dak Lak province by describing the characteristics of malaria cases; identifying potential risk factors for *Plasmodium falciparum* (Pf) and *Plasmodium vivax* (Pv) infection among reported cases; determining malaria clusters, and the temporal and spatial distributions of these cases between 2017 and 2022.

**Methods:**

Monthly district-level passive surveillance data on reported malaria cases from Dak Lak Center for Disease Control were analyzed using logistic regression, time series decomposition, negative binomial time series generalized linear model and spatial analyses.

**Results:**

Over the 6-year period, there were 2147 malaria cases reported in Dak Lak province, of which 58.2% were indigenous and 39.1% were imported. Pf was the dominant malaria parasite. Our findings revealed a considerable decline with malaria cases approaching zero since 2021. Malaria infections peaked in the late rainy season in October and November. Temperature and rainfall were most strongly associated with malaria case numbers at positive lags. The number of cases in a given month was highly dependent on the previous month’s cases. Gender, ethnicity, and living in higher endemicity areas were associated with having Pf and Pv infection. There were cold spots in southwest Dak Lak province while no hotspot areas were identified.

**Conclusions:**

Despite a dramatic reduction in malaria cases, social behavior change communication and preventive interventions should be maintained, especially for high-risk groups including men, ethnic minorities (H Mông in specific), people living in high transmission risk areas and mobile populations between provinces and across the Vietnam-Cambodia border. These need to be implemented ahead of the peak malaria season. Our findings, along with further operational research, are crucial to guide future elimination plans and resource allocation.

## Introduction

Globally, malaria remains a significant public health challenge, with an estimated 263 million cases and 597,000 deaths reported worldwide in 2023 [1]. However, over the past three decades, Vietnam has made tremendous progress in reducing mortality and morbidity associated with malaria [2]. The country’s targets are to eliminate *Plasmodium falciparum* by 2023 and achieve nationwide malaria elimination by 2030 [3, 4], with residual transmission now limited to a few provinces in the central region, in particular Dak Lak and Khanh Hoa [5]. However, several remaining challenges threaten these targets, including forest malaria transmission, seasonal and hard-to-reach populations, emerging drug resistance, and financial sustainability [3, 4, 6–8]. Additionally, imported malaria infections require particular attention since these cases are difficult to monitor, resulting in resurgences in areas that previously reported no cases [9, 10].

The Central Highlands represents a priority endemic region for targeted malaria control and elimination programs in Vietnam [2, 3, 6]. A previous study highlighted that hilly and forested areas in this region had hidden human malaria reservoirs with asymptomatic and submicroscopic malaria infections that could jeopardize elimination [11]. This region has also consistently demonstrated higher malaria risk compared to other areas in Vietnam [12].

Dak Lak province, located in the Central Highlands, has among the highest malaria burden in Vietnam [13]. Our aims in this study were to describe the characteristics of malaria cases; identify potential risk factors for *Plasmodium falciparum* (Pf) and *Plasmodium vivax* (Pv) infection among reported cases; determine malaria clusters; and the temporal and spatial distributions of these cases in Dak Lak province between 2017 and 2022. With current limited resources, identifying focal areas for better malaria prevention and control is crucial for informing evidence-based, targeted intervention strategies in this specific province and contributing to Vietnam’s broader malaria elimination goals.

## Methods

**Fig. 1:**
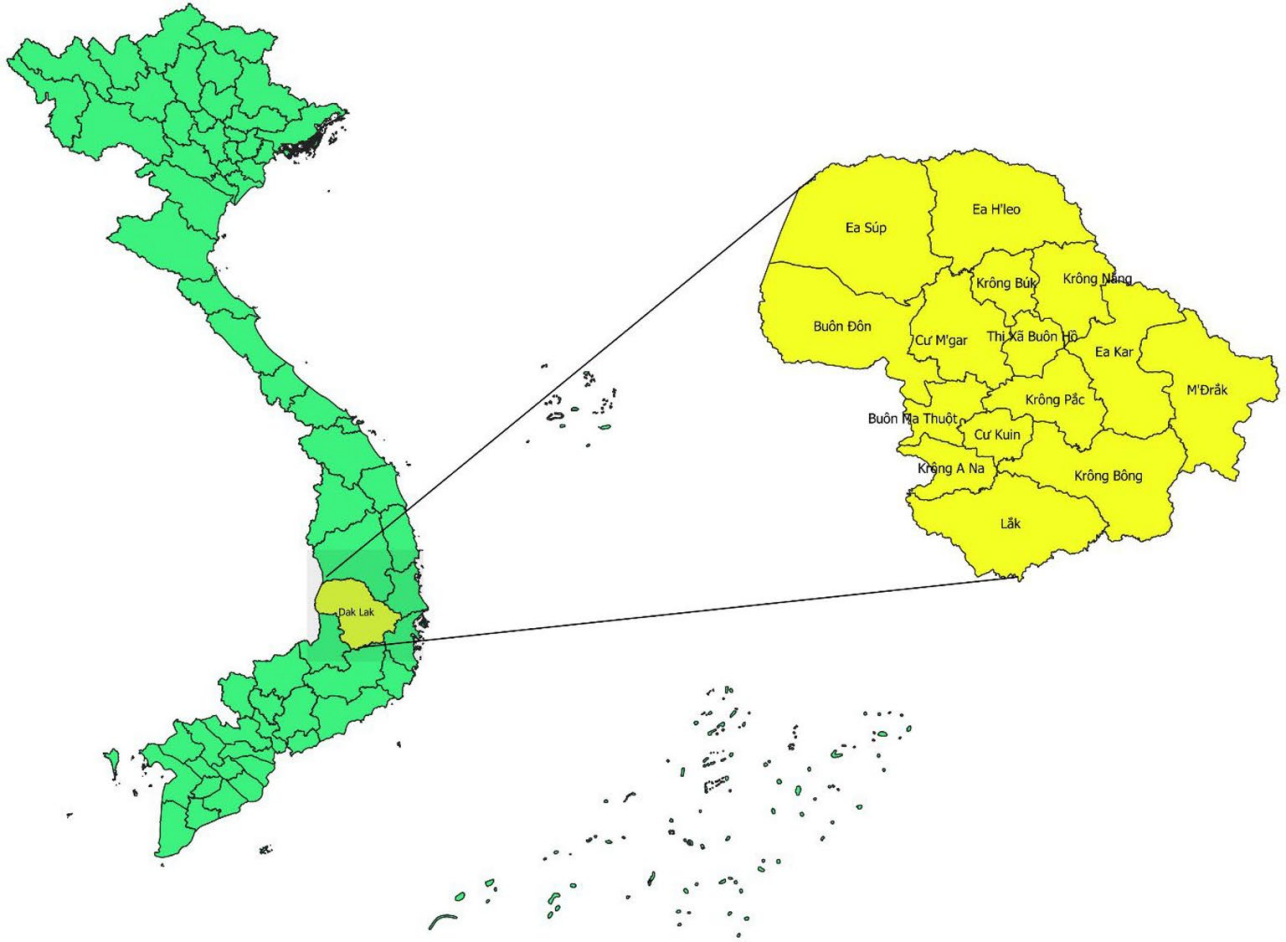
Map of Vietnam and Dak Lak province

This is a retrospective study using the secondary data sources of passive surveillance within Vietnam’s National Malaria Control Program. Monthly data on reported malaria cases from January 2017 to December 2022 were obtained from the Center for Diseases Control (CDC) of Dak Lak province, Vietnam. Climatic data, including average monthly temperature (°C) and total monthly rainfall (mm), were retrieved from the Dak Lak Meteorological Department. Population data was utilized from the reports of the Dak Lak Statistic Yearbook published by the Provincial Statistics Office. Dak Lak province is in the Central Highlands of Vietnam. Figure 1 shows the extent prior to merging with Phu Yen Province on 12th June 2025.

### Statistical analysis

All data was entered into Microsoft Excel for data management and transferred to R Studio version 2024.12.1 for statistical analysis. Logistic regression was used to identify risk factors for Pf malaria infection and Pv infections among reported malaria cases. Bivariate risk factors associations were sought for each gender, age groups, ethnic group, migrant status and residence in malaria endemicity areas. In our analysis, we began with a full model including all covariates and relevant variables and interaction, thereby minimizing the risk of omitting important predictors. Those with p < 0.05 were then included in stepwise, automatic, backward-elimination, unconditional logistic regression models based on the Akaike Information Criterion (AIC). This approach was used to identify a parsimonious model that balances goodness-of-fit and model complexity. Risk factors with p-value less than 0.05 were retained in the final multivariable regression models, with results presented as odds ratios (ORs) and 95% confidence intervals (CIs).

The malaria endemicity areas were stratified as defined in the National Strategic Plan on Malaria Control and Elimination 2021–2025 [3]. Clinical malaria cases were defined as cases diagnosed based on clinical symptoms without laboratory confirmation. These cases were excluded from species-specific regression analyses due to the absence of species classification. However, they were included in overall malaria counts for temporal and spatial analyses to capture the total burden of disease.

### Temporal analysis

Monthly case counts were compiled into a time series dataset aligned by month and year. Initial exploration included plotting the monthly total malaria cases by species over time using the *ggplot2* package to examine general trends and seasonal patterns.

Time series analyses were performed using Seasonal-Trend decomposition using Loess (STL) to separate malaria case counts into trend, seasonal, and remainder components. To assess stationarity, the Augmented Dickey-Fuller (ADF) test was applied (*adf* function). A p-value less than 0.05 was interpreted as evidence of stationarity. To examine lagged associations between climatic factors and malaria incidence, cross-correlation functions (CCFs) were computed (*ccf* function). CCFs between malaria cases and temperature, as well as rainfall, were calculated with 1-month lag of up to ± 12 months. Then we checked the dispersion and verified that the data consisted of discrete count values with substantial overdispersion (dispersion = 29.3). Next, we applied a negative binomial time series generalized linear model (tsGLM) using the *tscount* package (*tsglm* function) in R Studio to account for overdispersion and the count nature of malaria cases. The model incorporated one autoregressive term (*past_obs* = *1*) and one seasonal component at lag 12 (*past_mean* = *12*), to capture short-term and annual temporal dependence. Rainfall and temperature were included as external regressors (*xreg*) based on prior cross-correlation findings indicating delayed effects on malaria transmission. Model performance was also assessed using the AIC, Bayesian Information Criterion (BIC), and visual inspection of fitted versus observed series to select the best model for forecasting. The model structure was defined as:

$${\overline{\mathrm{Y}}}_{{\mathrm{t}}} \, \sim \,{\mathrm{nb}}\,\left( {\upmu_{{\mathrm{t}}} ,\upsigma^{{2}} } \right)$$

months, suggesting that increased temperature

$$\upmu_{{\mathrm{t}}} \, = \,\upbeta_{0} \, + \,\upbeta_{1} {\overline{\mathrm{Y}}}_{{{\mathrm{t}}\, - \,1}} \, + \,\upalpha_{12} {\overline{\mathrm{Y}}}_{{{\mathrm{t}}\, - \,12}} \, + \,\upeta_{1} {\mathrm{Rainfall}}_{{\mathrm{t}}} \, + \,\upeta_{2} {\mathrm{Temperature}}_{{\mathrm{t}}}$$

where Y_t_ is the malaria case count at time *t*, β_1_ captures short-term autocorrelation (past_obs = 1), α_12_ represents the yearly seasonal component (past_mean = 12), and η_1_, η_2_ denote the effects of rainfall and temperature. Parameter estimates were obtained by maximum likelihood, and 95% confidence intervals were derived using the normal approximation. Forecasts were generated for the subsequent 36 months under the simplifying assumption of average climatic conditions in Dak Lak province. Specifically, rainfall and temperature were held constant at their observed mean values over the study period due to the absence of reliable long-term climate projections. Forecasts were presented with 95% prediction intervals.

### Spatial analysis

Annual incidence per 1,000 population was calculated for each district. Choropleth maps were produced using *ggplot2* in R Studio. The Global Moran’s I statistic was calculated to evaluate the overall spatial autocorrelation of malaria incidence across districts in Dak Lak province with p < 0.05 considered statistically significant. To identify local clusters of high or low incidence, we applied local Getis-Ord Gi* statistics, which measure local spatial association for each district. These spatial analyses were performed using the *tmap*, *spdep* and *sf* packages. Districts were classified as hotspots (clusters of high incidence), cold spots (clusters of low incidence), or not significant based on Z-scores and p-values (α = 0.05). The results were visualized with district-level shapefiles projected to UTM Zone 48 N (EPSG:32648) to ensure spatial accuracy.

## Results

In total, 2147 malaria cases were reported in Dak Lak province between 2017 and 2022, including 1487 Pf cases and 561 Pv cases.

Of these total cases, 58.2% were indigenous infections (Table 1), 39.1% were imported and the remainder were unclassified. Amongst the imported cases, 54 (6.4%) infections were from Cambodia and located at the Vietnam-Cambodia border. One case was from Angola, Africa. The majority of infected cased were male (88.3%) and belonged to the 18–59 age group (89.1%). Malaria cases were reported in 20 ethnic groups, of which, Kinh, Ê Đê and H Mông accounted for the most (68.5%). 5% of cases were migrants.

**Table 1:** Characteristics of malaria cases

Characteristics	Indigenous N = 1250 N (%)	Imported N = 839 N (%)	Unclassified N = 58 N (%)	All N = 2147 N (%)
Gender				
Male	1090 (87.2)	758 (90.3)	47 (81.0)	1895 (88.3)
Female	160 (12.8)	81 (9.7)	11 (19.0)	252 (11.7)
Age groups				
Under 5 years	12 (0.9)	5 (0.6)	1 (1.7)	18 (0.9)
6–17 years	122 (9.8)	43 (5.1)	6 (10.3)	171 (7.9)
18–59 years	1092 (87.5)	775 (92.4)	44 (75.9)	1911 (89.1)
60 years or above	23 (1.8)	16 (1.9)	7 (12.1)	46 (2.1)
Ethnic groups				
Kinh	306 (24.5)	289 (34.4)	14 (24.1)	609 (28.4)
Ê Đê	275 (22.0)	215 (25.6)	17 (29.3)	507 (23.6)
H Mông	256 (20.5)	97 (11.6)	1 (1.7)	354 (16.5)
Others	413 (33.0)	238 (28.4)	26 (44.9)	677 (31.5)
Migrant				
Yes	1 (0.1)	102 (12.2)	0 (0.0)	103 (4.8)
No	1245 (99.9)	734 (87.8)	55 (100.0)	2034 (95.2)
Diagnostic results				
Clinical malaria	1 (0.1)	18 (2.2)	51 (87.9)	70 (3.26)
Mixed infection	16 (1.3)	12 (1.4)	0	28 (1.3)
Pf malaria	924 (73.9)	559 (66.6)	4 (6.9)	1487 (69.26)
Pv malaria	309 (24.7)	249 (29.7)	3 (5.2)	561 (26.13)
P. malariae malaria	0	1 (0.1)	0	1 (0.05)
Severeity				
Yes	8 (0.6)	2 (0.2)	0	10 (0.5)
No	1242 (99.4)	837 (99.8)	58 (100.0)	2137 (99.5)
Malaria endemicity area (Missing = 167)				
High transmission	482 (38.9)	94 (13.5)	14 (31.8)	590 (29.8)
Moderate transmission	602 (48.5)	300 (43.1)	11 (25.0)	913 (46.1)
Low transmission	149 (12.0)	222 (31.9)	13 (29.6)	384 (19.4)
Potential transmission	7 (0.6)	80 (11.5)	6 (13.6)	93 (4.7)
Type of patient (Missing = 22)				
In patient	842 (67.4)	552 (66.9)	38 (76.0)	1432 (67.4)
Outpatient	408 (32.6)	273 (33.1)	12 (24.0)	693 (32.6)
Type of treatment facility				
Public	1196 (95.7)	727 (86.8)	48 (85.7)	1971 (91.9)
Private	54 (4.3)	111 (13.2)	8 (14.3)	173 (8.1)

Pf was found in 70% and Pv in 26%. Ten cases were severe with 9 Pf cases and 1 Pv case. Mixed infections of Pf and Pv accounted for only 1.3%. One case had *P. malariae*.

### Risk factors

In bivariate logistic regression, among the reported malaria cases; being male, being H Mông, and living in higher endemicity areas were associated with higher odds of having Pf infection versus Pv infection (Table 2). Specifically, males had 49% higher odds of having Pf infection rather than Pv infection compared to female cases (p < 0.01). Individuals from the H Mông ethnic group had more than twice the odds of having Pf versus Pv infection compared to the Kinh group (p < 0.001). People living in high, moderate or low transmission areas had significantly higher odds of having Pf infection rather than Pv infection compared to people living in areas at risk of malaria re-introduction (p < 0.01).

**Table 2:** Bivariate and multivariable logistic regression models of risk factors for Pf and Pv infection among reported malaria cases

Risk factors	Pf malaria infection (n = 1483)			Pv malaria infection (n = 557)		
	Total n (%)	Bivariate ORs (95% CI)	Adjusted ORs (95% CI)	Total n (%)	Bivariate ORs (95% CI)	Adjusted ORs (95% CI)
Gender						
Male	1330	1.4 (1.1–1.9) **	1.5 (1.1–1.9) **	477	0.7 (0.5–0.9) *	0.7 (0.5–0.9) *
Female	153	Ref.	Ref.	80	Ref.	Ref.
Ethnic groups						
H Mông	297 (20.0)	2.5 (1.8–3.6) ***	2.1 (1.5–3.0) ***	52 (9.3)	0.4 (0.3–0.6) ***	0.5 (0.4–0.7) ***
Ê Đê	332 (22.4)	1.0 (0.8–1.3)	0.9 (0.7–1.2)	149 (26.8)	1.1 (0.8–1.4)	1.2 (0.9–1.6)
Others	453 (30.6)	1.1 (0.9–1.4)	1.0 (0.8–1.3)	188 (33.7)	1.0 (0.8–1.3)	1.1 (0.8–1.4)
Kinh	401 (27.0)	Ref.	Ref.	168 (30.2)	Ref.	Ref.
Malaria endemicity area						
High transmission risk	441 (29.7)	3.1 (1.9–4.9) ***	2.6 (1.6–4.2) ***	130 (23.3)	0.4 (0.2–0.6) ***	0.4 (0.3–0.7) **
Moderate transmission risk	658 (44.4)	2.5 (1.6–3.9) ***	2.3 (1.4–3.6) ***	228 (40.9)	0.5 (0.3–0.7) **	0.5 (0.3–0.8) **
Low transmission risk	257 (17.3)	2.1 (1.3–3.4) **	2.2 (1.3–3.5) **	110 (19.7)	0.6 (0.4–0.9) *	0.6 (0.3–0.9) *
Unknown	83 (5.6)	1.2 (0.7–2.1)	1.2 (0.7–2.1)	53 (9.5)	0.8 (0.5–1.4)	0.8 (0.4–1.4)
Areas at risk of malaria re-introduction	44 (3.0)	Ref.	Ref.	36 (6.5)	Ref.	Ref.

*Ref* Reference, *ORs* Odd Ratios, *CI* Confidence Interval, ***p < 0.001, **p < 0.01, *p < 0.5

In stepwise logistic regression, males had significantly lower odds of having Pv infection over Pf infection compared to females (OR = 0.7, 95% CI 0.5–0.9, p = 0.012). H Mông ethnicity was significantly associated with lower odds of having Pv infection compared to Kinh (OR = 0.5, 95% CI 0.4–0.7, p < 0.001). Compared to areas at risk of malaria re-introduction, patients living in high transmission areas had significantly lower odds of Pv over Pf infection (OR = 0.4, 95% CI 0.3–0.7, p = 0.001). Patients living in low or moderate transmission areas had significantly lower odds of Pv over Pf infection (OR = 0.6, 95% CI 0.3–0.9, p = 0.02 and OR = 0.5, 95% CI 0.3–0.8, p = 0.004, respectively) comparing to patients living in areas at risk of malaria re-introduction.

### Temporal analysis of malaria cases

**Fig. 2:**
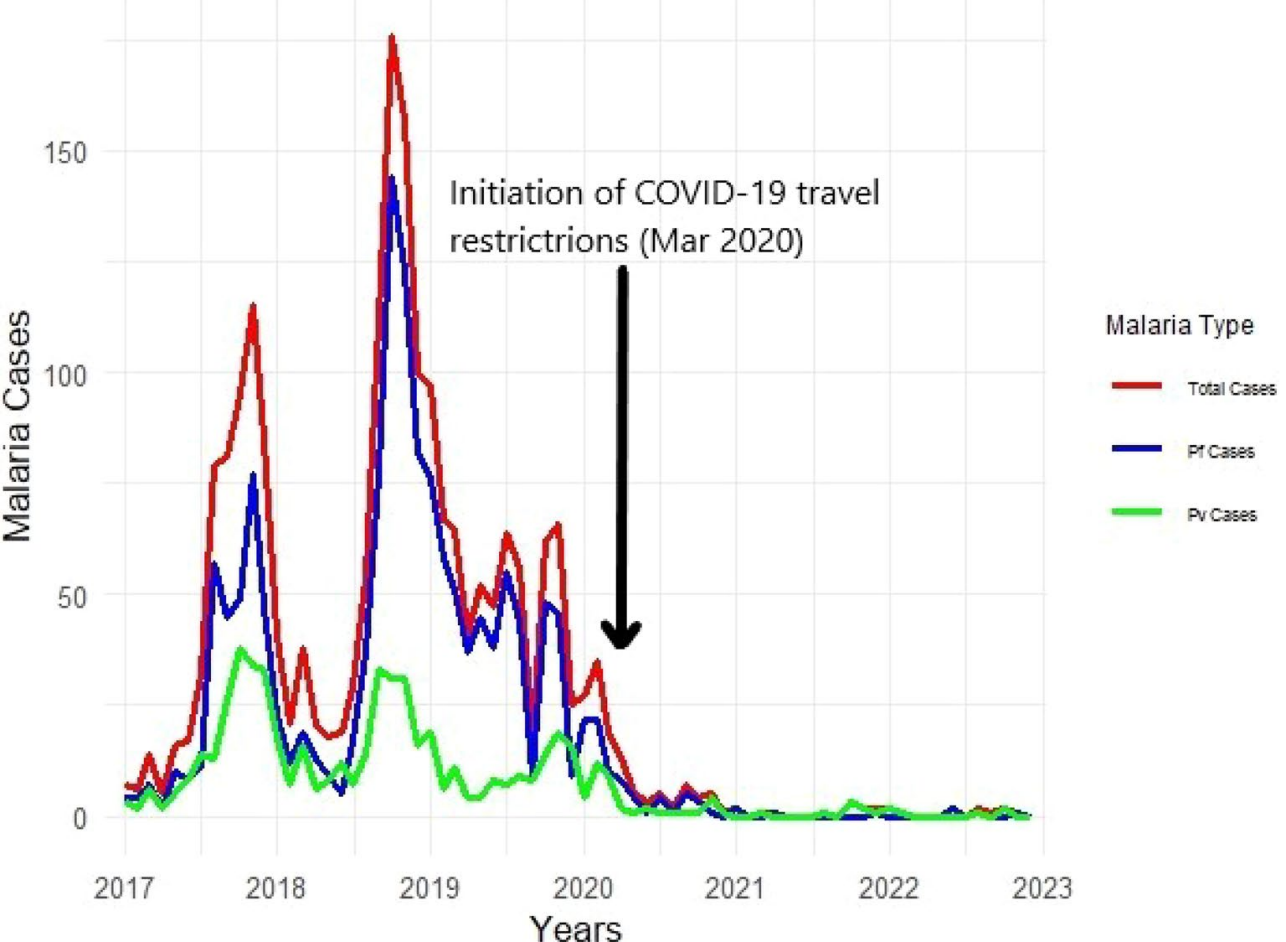
Total monthly malaria cases in Dak Lak from 2017 to 2022

**Fig. 3:**
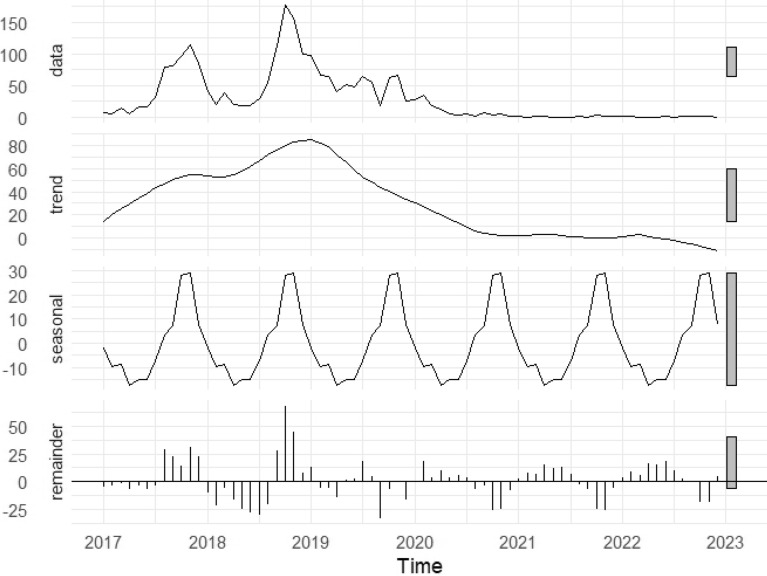
Seasonal-trend decomposition of monthly malaria cases, 2017–2022. Panels show the observed data (top) of the monthly number of reported malaria cases, long-term trend (second panel) representing the long-term underlying pattern in case counts over time, seasonal pattern (third panel) reflecting the recurring within-year variation in malaria transmission, expressed as deviations from the overall trend, and residual variation (bottom panel) capturing the irregular fluctuations not explained by the trend or seasonal components. All y-axes represent malaria case counts or relative deviations in case counts

There were peaks in cases in 2017, 2018 and 2019. The year with the highest number of cases was 2018, with 786. After the first half of 2020, numbers of cases were very low (Fig. 2). The time series decomposition in Fig. 3 highlighted a clear seasonal pattern in malaria cases, with consistent annual peaks corresponding to the transmission season. The trend component showed an increase in cases from 2017, peaking in late 2019, followed by a sharp decline, with cases approaching zero by 2021. The remainder component indicated some irregular fluctuations, particularly during the period of rising cases, but these became minimal as overall case numbers declined.

**Fig. 4:**
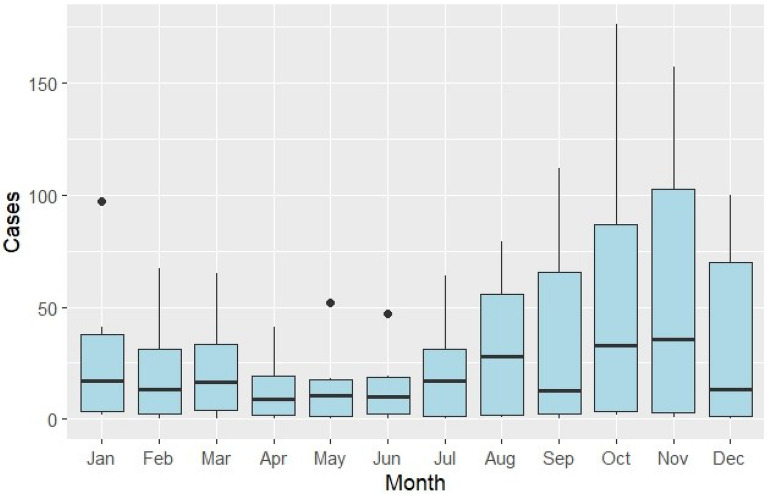
Annual cases by month during 2017–2022

Although the Augmented Dickey-Fuller test indicated that the malaria case time series was stationary (Dickey-Fuller = −3.78, lag order = 4, p = 0.024), visual inspection of the STL decomposition suggested the presence of seasonal variation. The majority of cases were reported during the second half of each year (Fig. 4), peaking in October–November.

**Fig. 5:**
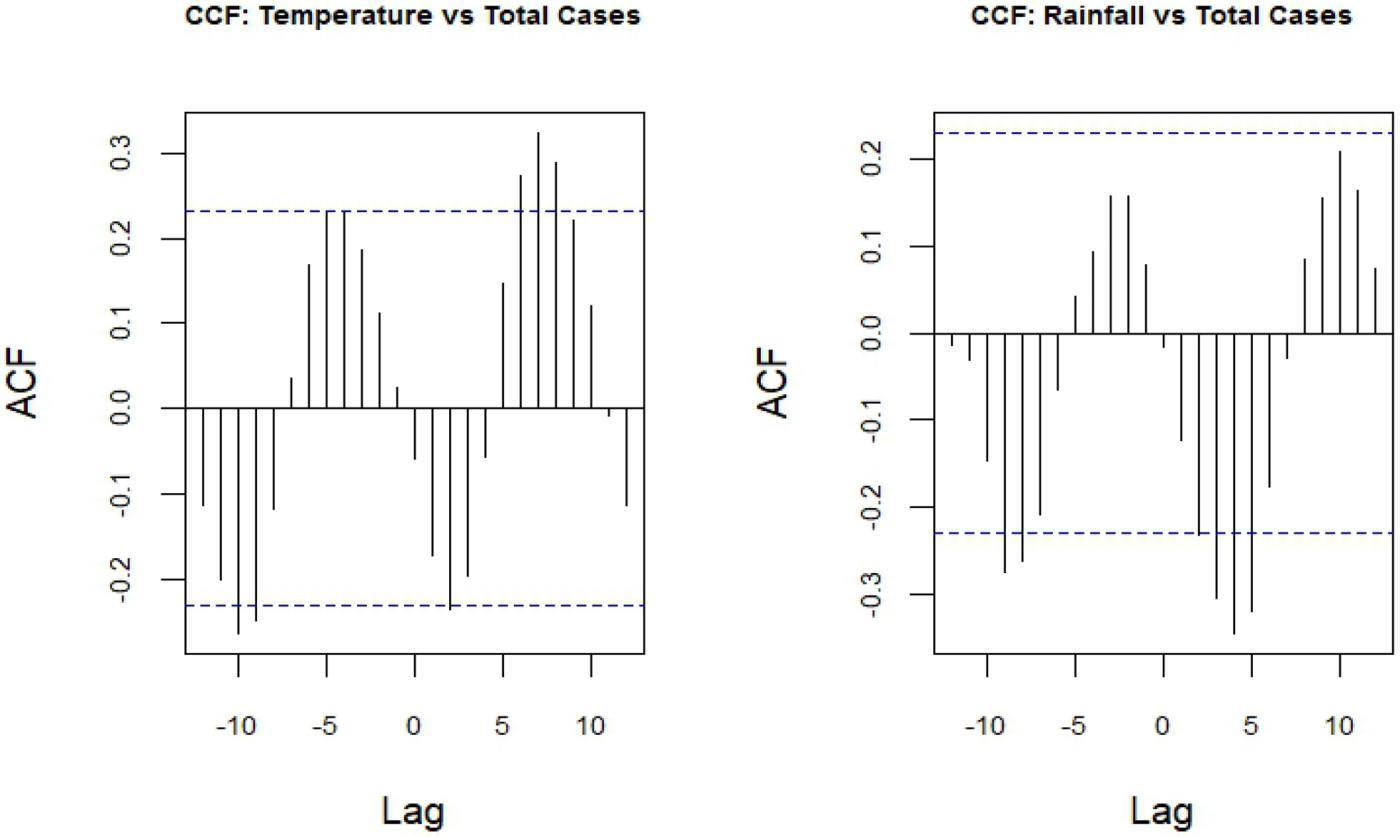
Cross-correlation analyses between malaria cases and temperature and rainfall. *ACF* autocorrelation function. Lag in months

Cross-correlation analyses indicated that both temperature and rainfall were most strongly associated with malaria case numbers at positive lags (Fig. 5). Specifically, temperature showed the strongest correlations at lags of approximately +6 to +8 months, suggesting that increased temperature may contribute to higher malaria transmission after a delay of 6–8 months. While rainfall showed negative correlations at lags of +3 to +5, this suggests that higher rainfall levels may be associated with lower malaria case numbers 3–5 months later.

The final negative binomial time series model was fitted with lagged rainfall (3 months) and temperature (6 months) as covariates since it substantially improved model performance with lowest AIC of 450.4 compared with the baseline model without climatic variables or with alternative lag specifications. It was expressed as:

$${\overline{\mathrm{Y}}}_{{\mathrm{t}}} \, \sim \,{\mathrm{nb}}\,\left( {\upmu_{{\mathrm{t}}} ,\upsigma^{{2}} } \right)$$

$$\upmu_{{\mathrm{t}}} \, = \,\upbeta_{0} \, + \,\upbeta_{1} {\overline{\mathrm{Y}}}_{{{\mathrm{t}}\, - \,1}} \, + \,\upalpha_{12} {\overline{\mathrm{Y}}}_{{{\mathrm{t}}\, - \,12}} \, + \,\upeta_{1} {\mathrm{Rainfall}}_{{{\mathrm{t}}\, - \,3}} \, + \,\upeta_{2} {\mathrm{Temperature}}_{{{\mathrm{t}}\, - \,6}}$$

**Fig. 6:**
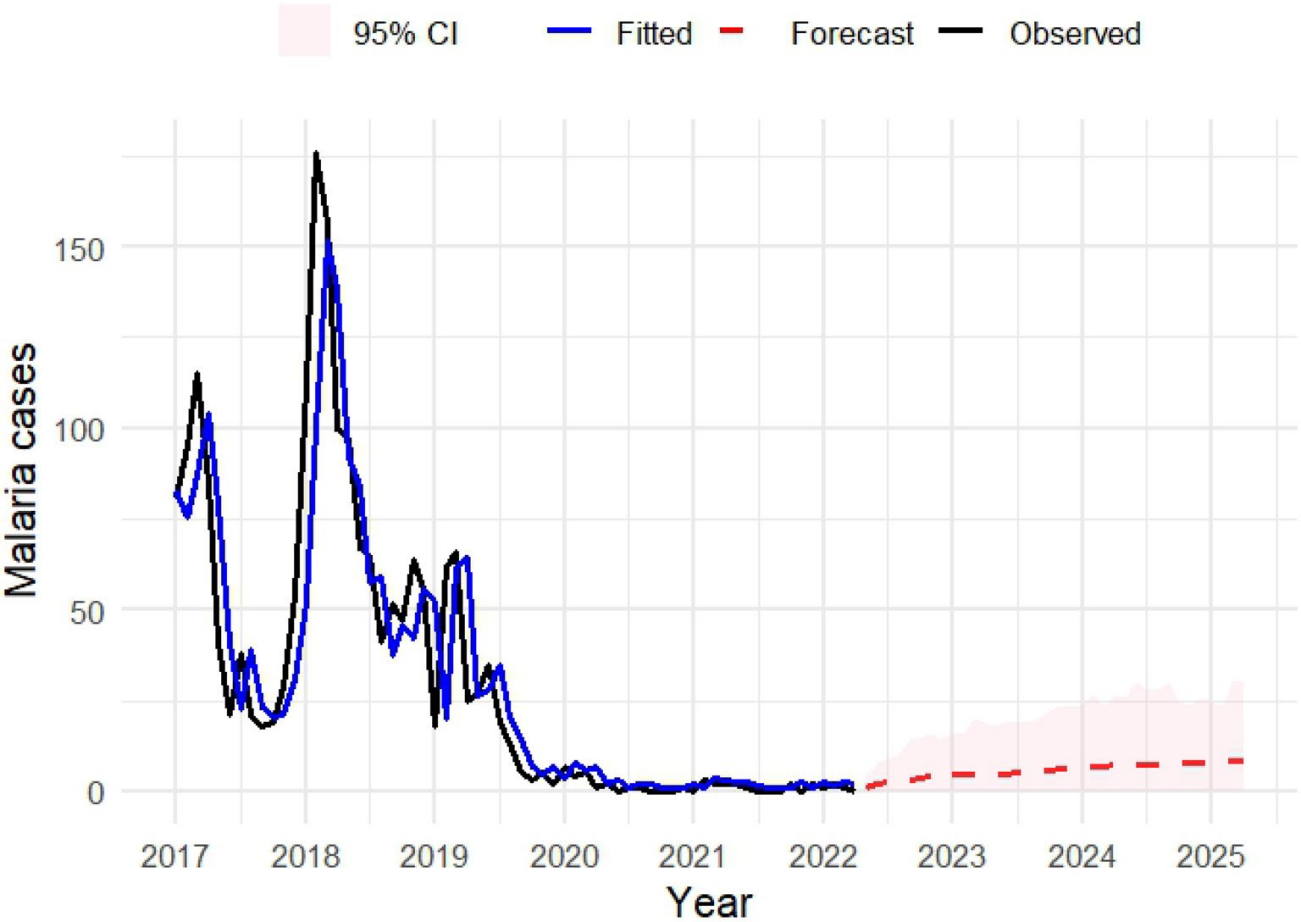
Forecasting model for malaria cases with lagged temperature and rainfall in the next 36 months

The model indicated a strong autoregressive effect (β1 = 0.914, 95% CI 0.7–1.1), suggesting that the number of cases in a given month is highly dependent on the previous month’s cases. The seasonal effect (α_12_ = 0.083) and both lagged covariates (rainfall η₁ = 0.00025; temperature η₂ = −0.0027, p > 0.05) were not statistically significant, with CI including zero. The overdispersion parameter (σ^2^ = 0.276) confirms extra-Poisson variability. The forecast suggests a gradual increase in monthly and yearly case counts under average climatic conditions. However, the wide confidence intervals indicate substantial uncertainty, reflecting both overdispersion and temporal variability in the model (Fig. 6).

### Spatial analysis of malaria cases

**Fig. 7:**
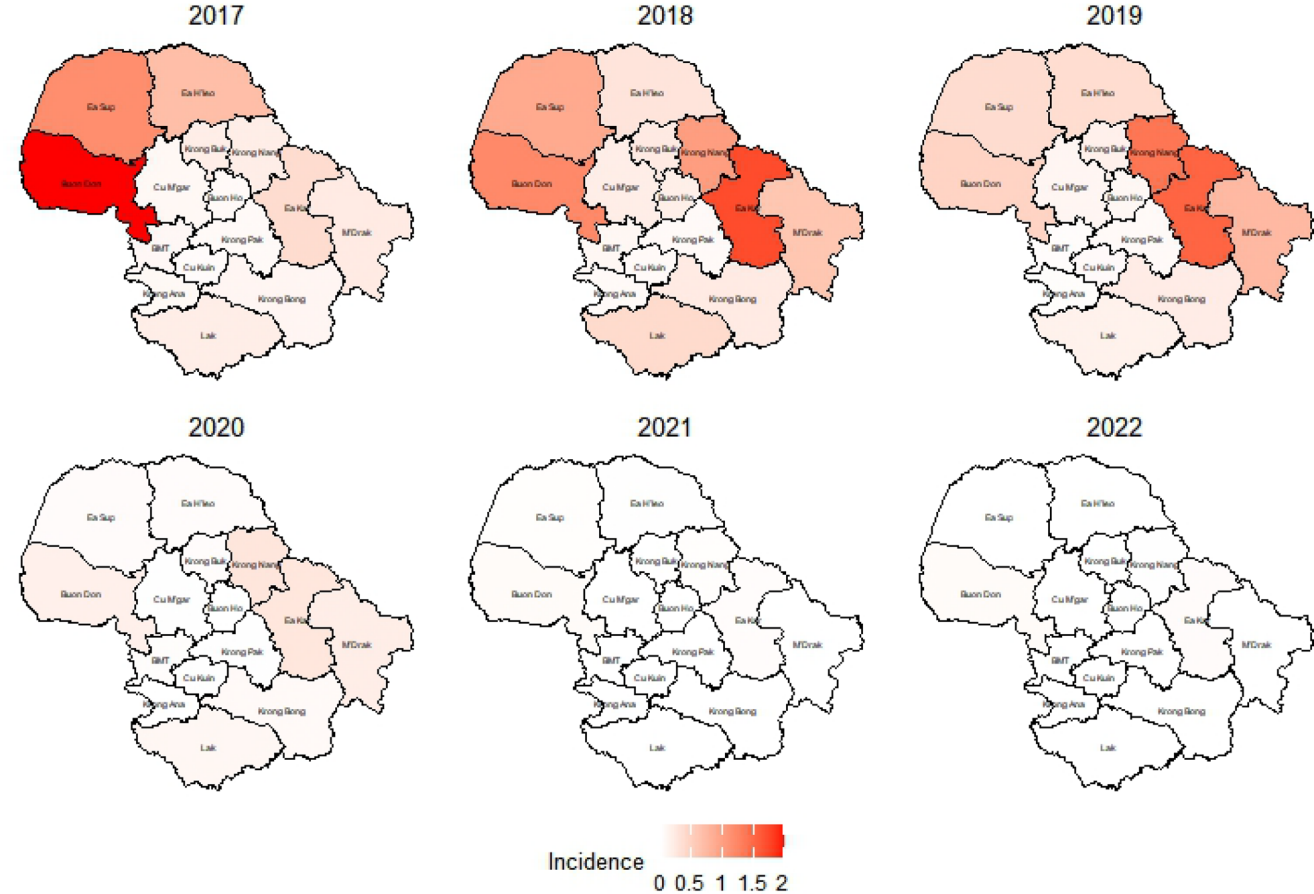
Malaria incidence per 1000 population by districts in Dak Lak over the year

The highest malaria incidence rates in Dak Lak province were in Buon Don district in 2017 (2.1 cases per 1,000 person-years) and Ea Kar district in 2018 (1.7 cases per 1,000 person-years) and 2019 (1.5 cases per 1000 person-years) (Fig. 7).

**Fig. 8:**
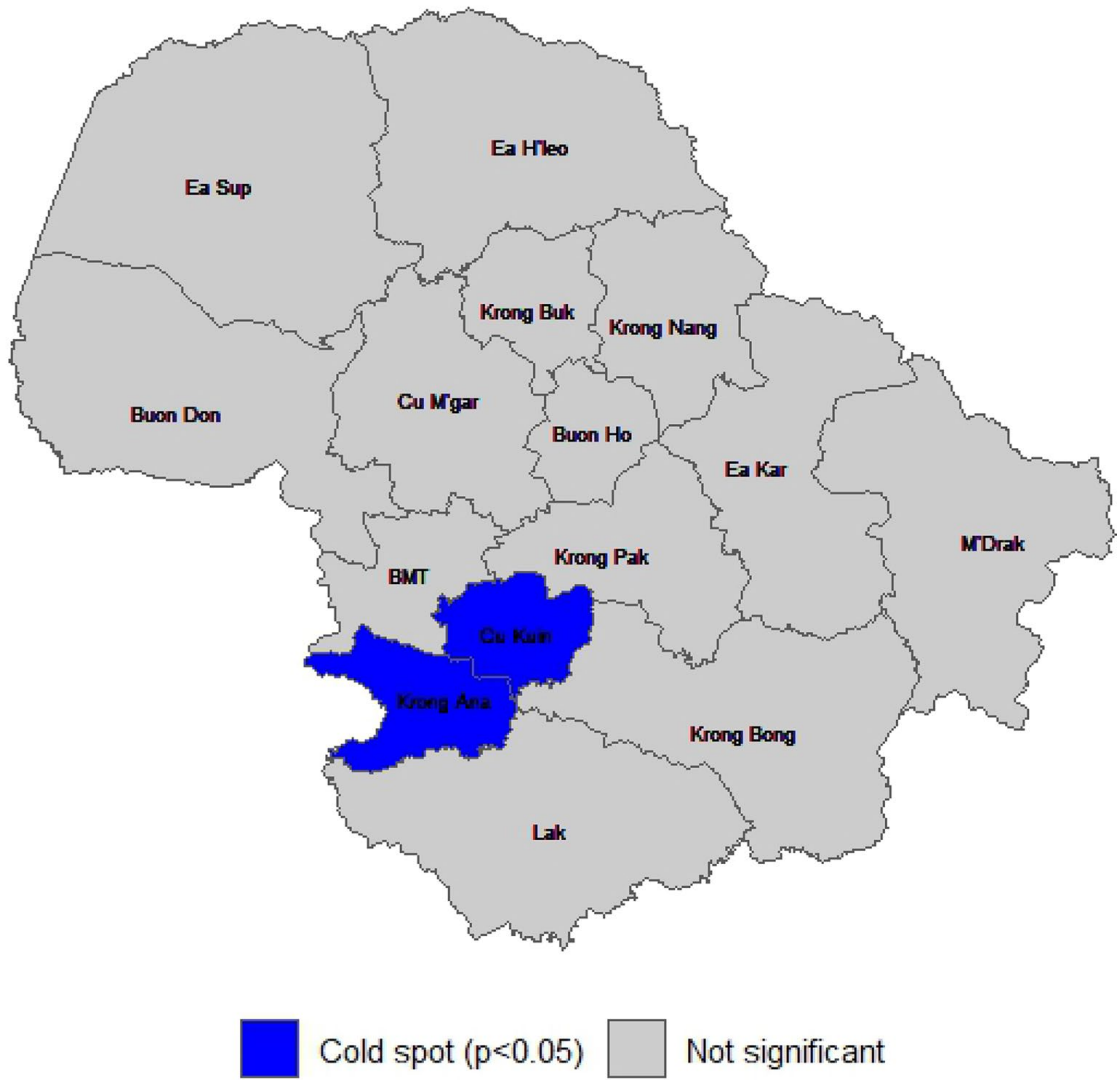
Hotspot analysis mapping in Dak Lak province

Local Gi* hotspot analysis (Fig. 8) revealed statistically significant cold spots of malaria incidence in Krong Ana and Cu Kuin districts in the southwest of the province, where incidence was consistently lower than neighboring districts (p < 0.05). No statistically significant hotspots were identified. This finding aligned with the Global Moran’s I result, which showed a small but significant positive spatial autocorrelation (Moran’s I = 0.0581, p = 0.0006), suggesting that malaria incidence was weak but significantly clustered in space.

## Discussion

This study found a dramatic decline in reported malaria cases in an endemic province over a 6-year period from 2017 to 2022. This trend aligned with broader progress towards elimination across Vietnam where the National Malaria Control Program achieved a remarkable reduction in deaths and cases during its early and later implementation phases [4, 14]. In a recent report, 35 provinces of Vietnam had been certified as malaria-free by 2020 [7, 15]. Vietnam’s progress towards malaria elimination represents one of the regional public health achievements [4, 16]. However, this near-elimination in a focal point such as Dak Lak must be interpreted cautiously, as several factors could contribute to the observed decline beyond true transmission reduction. The COVID-19 pandemic significantly disrupted malaria surveillance and reporting system in the GMS including Vietnam, with countries reporting substantial decreases in reported malaria cases during 2020–2021 that likely reflect surveillance artefacts rather than genuine elimination [7, 17]. Particularly, Vietnam implemented nationwide COVID-19-related restrictions of public movement and travel in 2020–2021. These restrictions led to decreased testing rates, reduced routine field-based surveillance activities or healthcare-seeking behavior, creating significant surveillance gaps during the period when Dak Lak’s cases dropped to near-zero levels [7, 17]. However, the extent of this disruption could not be quantified due to the lack of detailed surveillance indicators, such as testing rates or reporting completeness.

Furthermore, the nationwide travel bans likely reduced cross-border movement and, consequently, the number of imported cases from Cambodia—previously a major transmission source as shown in this study. While this decline in imported cases represents a genuine reduction, cross-border movement has since resumed with potential for re-importation. However, case numbers in Cambodia have greatly declined and the country (as also in Lao PDR and eastern Thailand) is nearing elimination, so this risk is now very low. However, transmission in the Western Mekong Region along the Thai-Myanmar border has increased substantially since 2021 [18] with potential for spread further east. Malaria endemic areas in Thailand have been shown using mobile phone data to be poorly connected to each other [19] although movement patterns may change over time. Population movements pose multiple challenges for elimination programs, including threats to antimalarial drug efficacy through the introduction of drug-resistant strains and the difficulty of ensuring complete case investigation and contact tracing for mobile groups [7, 20]. These challenges highlight the importance of regional cooperation and coordinated elimination strategies across the GMS, following the World Health Organization’s framework for malaria elimination [21] and specific strategies for GMS [22].

The logistic regression analysis revealed several interconnected demographic and socioeconomic factors that are associated with Pf versus Pv infection among reported malaria cases. Male gender significantly increased the odds of infection, consistent with previous studies in Vietnam [7, 9, 20]. In our analysis, working-age individuals (18–59 years) were at highest risk, compared to other age groups in malaria patients. These associations likely reflect patterns of occupational exposure, as working-age males in Dak Lak province are the main group engaged in high-risk activities. This finding was aligned with other studies in Cambodia, Myanmar and Thailand [23–25]. In particular, forest work significantly increases contact with malaria vectors in forest-fringe environments [4, 6, 7], particularly for men [10, 20].

Ethnicity emerged as a particularly strong predictor of Pf infection, with H Mông individuals demonstrating twice the odds of Pf over Pv infection compared to Kinh populations (OR = 2.1, 95% CI 1.5–3.0). This elevated risk reflects a complex interaction between socioeconomic, geographic, and cultural factors. H Mông communities in Dak Lak province typically reside in remote, hilly, and forested areas with optimal ecological conditions for malaria transmission [10, 11], while simultaneously facing barriers to healthcare access and delays in treatment-seeking due to cultural beliefs and practices [6, 10, 16]. Additionally, their traditional housing structures and frequent engagement in forest-based economic activities create sustained exposure opportunities to infected vectors [10, 11]. The convergence of these risk factors in high malaria endemic areas creates synergistic effects that substantially amplify Pf transmission risk among these vulnerable populations.

Our findings also showed that patients living in high, moderate or low transmission areas had significantly lower odds of Pv infection compared to patients living in areas at risk of malaria re-introduction. Possible explanations could be that Pf, as the dominant parasite in Dak Lak province, somehow competed with Pv, or due to the differential effects of elimination measures on the two species [26]. However, Pv possesses several biological advantages that may contribute to its resilience relative to Pf. Unlike Pf, Pv can form dormant liver-stage parasites known as hypnozoites, which are capable of reactivating months after the initial infection and causing relapse without re-exposure [4]. Pv is also considered more tolerant of climatic and environmental changes [12]. Pv infections are more frequently asymptomatic compared to Pf [11], which might reduce case-seeking behavior and complicate case detection. Together, these biological characteristics explain why the relative proportion of Pv and Pf can shift disproportionately over time, a pattern that has been documented in previous studies [4, 7], and this carries important implications for malaria elimination programs.

The temporal analysis identified strong seasonal transmission patterns, with case numbers consistently peaking during October and November across the study period. This seasonality is earlier in the year than findings of a national review in Vietnam indicating that the malaria transmission in Southern and Central provinces has the seasonal peak in December–February [6] but similar to another study in Central Vietnam [27]. However, it aligns reasonably with biological mechanisms driving the transmission in the Central Highland areas, including optimal vector breeding cycles during the late rainy season when temperature and humid conditions favour mosquito development. These findings are also consistent with an analysis across 15 provinces in Central Vietnam between 2018 and 2022, which demonstrated similar year-end peaks for both Pf and Pv infections [12]. It implies that intervention activities such as knowledge and behavior change campaigns for high-risk populations should be conducted ahead of the peak season of malaria transmission.

Cross-correlation analysis indicated complex relationships between climatic factors and malaria cases, characterized by significant lag effects. Increased monthly temperatures were associated with higher malaria cases at 6–8 month lags, consistent with the above-mentioned study across Central Vietnam [12], likely reflecting accelerated mosquito breeding and development cycles. Conversely, increased rainfall showed negative correlations with malaria cases at 3–5 month lags, which might be a counterintuitive finding. While moderate rainfall generally creates favorable environments for mosquito breeding, excessive precipitation can have detrimental effects on vector population by diluting breeding sites and flushing out mosquito larvae [28, 29]. Human behaviour also changes during heavy rainfall periods, such as reduced outdoors activities, leading to decreased human exposure [29]. Our forecasting model suggested a gradual small increase in malaria burden in the coming months; however, the projections should be interpreted with great caution due to large forecast uncertainty. This uncertainty simplifies real-world climatic variability and may not reflect the future environmental changes, heterogeneous malaria transmission across districts, and ongoing control interventions in Dak Lak, particularly in the context of malaria elimination. Potential predictors not included in this analysis, such as vector control interventions, healthcare access, and population mobility, may improve model performance and should be considered in future studies. Although rainfall and temperature have negligible short-term effects in the model, strengthening early warning systems and integrating climate and health data could improve forecasting accuracy and support timely public health responses. These findings also emphasize the need for maintaining and enhancing surveillance capacity, prevention, and control efforts, particularly in areas with persistent transmission even though the projected increases in malaria cases were very modest and associated with substantial uncertainty.

Spatial analysis identified Krong Ana and Cu Kuin districts as significant cold spots with consistently low malaria transmission, while no significant hot spots were detected during the study period. These spatial patterns may suggest successful malaria control at the district level; however, further investigation is warranted to identify specific factors contributing to these achievements. Potential explanations might include the occupational exposure, mobility patterns, reduced proximity to forested environments or fewer forest areas resulting in reduced transmission risk for vulnerable populations in these two districts. It is also possible that more effective implementation of control measures such as vector control, social behavior changes communication interventions and surveillance activities, played a role although detailed intervention data were not available in this study. Additionally, Dak Lak developed their own elimination roadmaps for every district that will be a good example for other provinces in Vietnam to move closer to the elimination target [6]. Understanding the determinants of low transmission in these cold spot areas could inform resource allocation and intervention strategies for other high-risk areas. Although the absence of hot spots could represent successful malaria control across the province, the use of district-level data may not reflect localized heterogeneity, and smaller-scale hotspots at the commune or village level may not be detected in this analysis. Therefore, the absence of hotspots does not indicate complete spatial homogeneity of risk. Future studies using higher-resolution spatial data are needed to better identify micro-level transmission patterns and to guide targeted interventions. Continued surveillance remains important component to detect potential re-establishment areas early, as suggested in another study in the Thailand-Myanmar border [30].

The findings of this study can be further interpreted within a One Health framework, which recognizes the interconnected roles of environmental, human, and biological factors in malaria transmission. In the Central Highlands of Vietnam, environmental conditions such as forest coverage and climate variability create suitable habitats for malaria vectors, while human behaviors, including forest-related occupations and population mobility, increase exposure to infected mosquitoes. At the same time, parasite characteristics, including species-specific transmission dynamics, contribute to the persistence of malaria in low-transmission settings. These interacting factors highlight that malaria elimination cannot rely solely on biomedical interventions but require coordinated, multi-sectoral approaches [31]. Strengthening collaboration between public health, environmental management, and local communities will be essential to address residual transmission and prevent resurgence in this region.

Several limitations should be acknowledged in interpreting our findings. Our analysis was constrained by the inherent limitations of passive surveillance systems. We did not take into account the impact from malaria control interventions, forest coverage, forest activities and population movements of the malaria cases that would influence transmission patterns as there was no suitable data available. We used backward stepwise selection for multivariate regression modelling that can increase risk for type 1 errors and exclusion of relevant but non-significant predictors. To mitigate these concerns, we forced all variables deemed highly relevant to remain in the model and considered epidemiological plausibility when interpreting results. We employed the stepwise model as an exploratory tool to guide model building rather than for causal inference. Future studies with confirmatory designs should consider Bayesian approaches to improve model stability and inference.

In addition, the dramatic reduction in malaria cases during 2020–2022 must be interpreted cautiously given the well-documented disruptions to the surveillance system during COVID-19. Finally, the inclusion of clinical malaria cases in temporal and spatial analyses may introduce uncertainty, as these cases were not laboratory-confirmed and a large proportion were unclassified by origin. This may lead to potential misclassification and could influence the observed trends and spatial patterns. However, excluding these cases could underestimate the overall malaria burden. Also, classification challenges for imported cases might have affected the accuracy of our transmission source analysis, particularly for mobile populations along the Vietnam-Cambodia border.

## Conclusion

In summary, Dak Lak province experienced a significant decreasing trend of reported malaria cases from 2017 to 2022, that mirrored the general trend of the whole of Vietnam and the eastern GMS. Interventions regarding social behavior change communication and malaria prevention and control activities should be implemented before the peak season of malaria transmission and focus more on males, ethnic minorities (H Mông in specific), people living in high transmission risk areas and mobile populations between provinces and the Vietnam-Cambodia border. Further operational research is required to generate knowledge for future elimination activities and interventions for these high-risk groups in foci areas and maintain the overall malaria program in the context of limited resources.

## Data Availability

The datasets used and/or analysed during the current study are available from the corresponding author on reasonable request.
